# Insights into Prokaryotic Community and Its Potential Functions in Nitrogen Metabolism in the Bay of Bengal, a Pronounced Oxygen Minimum Zone

**DOI:** 10.1128/spectrum.00892-21

**Published:** 2022-05-17

**Authors:** Bowei Gu, Jiaxing Liu, Shunyan Cheung, Ngai Hei Ernest Ho, Yehui Tan, Xiaomin Xia

**Affiliations:** a Key Laboratory of Tropical Marine Bio-resources and Ecology, South China Sea Institute of Oceanology, Chinese Academy of Sciences, Guangzhou, China; b University of Chinese Academy of Sciences, Beijing, China; c Southern Marine Science and Engineering Guangdong Laboratory (Guangzhou), Guangzhou, China; d Department of Ocean Science, The Hong Kong University of Science and Technology, Hong Kong, China; e Hong Kong Branch of Southern Marine Science and Engineering Guangdong Laboratory, The Hong Kong University of Science and Technology, Hong Kong, China; Technical University of Denmark

**Keywords:** oxygen minimum zones, Bay of Bengal, prokaryotic community composition, prokaryotic function

## Abstract

Ocean oxygen minimum zones (OMZs) around the global ocean are expanding both horizontally and vertically. Multiple studies have identified the significant influence of anoxic conditions (≤1 μM O_2_) on marine prokaryotic communities and biogeochemical cycling of elements. However, little attention has been paid to the expanding low-oxygen zones where the oxygen level is still above the anoxic level. Here, we studied the abundance and taxonomic and functional profiles of prokaryotic communities in the Bay of Bengal (BoB), where the oxygen concentration is barely above suboxic level (5 μM O_2_). We found the sinking of *Trichodesmium* into deep water was far more efficient than that of *Prochlorococcus*, suggesting *Trichodesmium* blooms might be an essential carbon and nitrogen source for the maintenance of the BoB OMZ. In addition to the shift in the prokaryotic community composition, the abundance of some functional genes also changed with the change of oxygen concentration. Compared to oxic (>60 μM O_2_) Tara Ocean and high-hypoxic (>20 to ≤60 μM O_2_) BoB samples, we found more SAR11-*nar* sequences (responsible for reducing nitrate to nitrite) in low-hypoxic (>5 to ≤20 μM O_2_) BoB waters. This suggested SAR11-*nar* genes would be more widespread due to the expansion of OMZs. It seems that the nitrite-N was not further reduced to nitrogen through denitrification but likely oxidized to nitrate by *Nitrospinae* in the BoB OMZ and then accumulated in the form of nitrate-N. However, the lack of N_2_ production in the BoB would change if the BoB OMZ became anoxic. Together, these results suggested that reduction of oxygen concentration and OMZ expansion may increase the use of nitrate by SAR11 and N_2_ production in the BoB.

**IMPORTANCE** Recognizing the prokaryotic community and its functions in hypoxic (>5 to ≤60 μM O_2_) environments before further expansion of OMZs is critical. We demonstrate the prokaryotic community and its potential functions in nitrogen metabolism in the Bay of Bengal (BoB), where oxygen concentration is barely above suboxic level. This study highlighted that *Trichodesmium* might be an essential carbon and nitrogen source in the maintenance of the BoB OMZ. Additionally, we suggest that the lack of N_2_ production in the BoB would change if the BoB OMZ became anoxic, and the expansion of OMZs in the global ocean may potentially increase the use of nitrate by SAR11.

## INTRODUCTION

Oxygen minimum zones (OMZs) are defined as subsurface oceanic regions (100- to 1,500-m depth) with low oxygen concentrations (≤20 μM) due to decomposition of sinking organic material as well as reduced ventilation (1, 2). The O_2_ concentration of suboxic OMZs is ≤5 μM, while anoxic OMZs have ≤10 nM O_2_ (3, 4). There are four permanent OMZs in the global ocean, including the eastern tropical North Pacific (ETNP), the eastern tropical South Pacific (ETSP), the Arabian Sea (AS), and the Bay of Bengal (BoB), all of which are hot spots of pivotal processes that mediate the biogeochemical cycles of elements, the flux of greenhouse gases (e.g., CH_4_ and N_2_O), and trace metal recycling in the ocean (5). Increasing stratification and enhanced biological oxygen demand caused by climate change and anthropogenic pollution are driving the expansion of these OMZs both horizontally and vertically (6–8).

Under suboxic/anoxic conditions, some microbes adapt to utilize nitrate as an electron acceptor (1, 9) due to its free energy of reduction being similar to that of oxygen. Hence, organic carbon mineralization in OMZs is fueled principally by the reduction of nitrate to nitrite. This process is carried out by a variety of microbes, including the abundant SAR11 (*Pelagibacteraceae*) clade (10). Moreover, nitrite can be further reduced to N_2_ by denitrification or anammox. These two processes are estimated to make 30% to 50% of the oceanic fixed-nitrogen (N) loss in OMZs (11, 12). Other biogeochemical processes mediated by microorganisms, such as sulfur cycling, can be detected in open-ocean OMZs. For example, sulfate reduction genes in particles and sulfur-oxidizing genes in the water column were detected (13), while other studies found that sulfur oxidation occurs in particles but not in the water (14, 15). Studies have pointed out that ongoing global expansion and intensification of OMZs may influence the microbial community compositions and functions and that the marine nutrient cycles and carbon budget may be altered, thus resulting in a higher loss of nitrogen and production of greenhouse gases (9, 16).

The BoB has a strong seasonality driven by the Asian monsoon system (17). Heavy rainfall and the associated river discharge in summer (June to September) cause a considerable drop in surface water salinity (18). This, together with increased surface water temperature, induces strong stratification of the water column, limiting nutrient flux from subsurface water to the ocean surface, therefore making the BoB an area of relatively low primary production compared to the Arabian Sea (19). The BoB does not span a broad region with absolute anoxia as the other three OMZs do, and no indication of nitrogen loss has been observed although oxygen levels are below 1 to 2 μM (20). Additionally, under the stratified and nitrogen-limited conditions, blooms of *Trichodesmium*, a common diazotrophic cyanobacterium, occur sporadically and abruptly in the BoB (21). This makes the BoB an ideal area to recognize the diversity and metabolic properties of the prokaryotic community in hypoxic waters. With the development of sequencing technology and data analysis methods, investigations on microbial diversity and related functional metabolism have become more accurate and detailed. For instance, the application of metagenomics and metatranscriptomics has produced generous information about prokaryotic diversity and metabolism function in the ETNP, ETSP, and Arabian Sea (5, 13, 14, 22–26). However, to our knowledge, the prokaryotic potential metabolism based on metagenomics in the BoB has not been reported. Several studies had revealed that abundant microbes were involved in nitrogen and sulfur metabolism pathways based on quantitative PCR of functional genes or Tax4Fun prediction in the BoB (20, 27). However, the functions predicted from taxonomies (inferred by 16S rRNA) are inaccurate, especially in nonhuman samples (28). To understand the significant taxa which participate in biogeochemical processes, metagenomic techniques need to be applied to provide an accurate and comprehensive analysis of the functional profiles of the microbes in the BoB OMZ. This work utilized both amplicon sequencing and metagenomic approaches to comprehensively recognize the taxonomic and functional diversity of prokaryotes in hypoxic waters. Additionally, we compared the metagenomic data set from the OMZ layer in the BoB (500 m) and that from the oxygenated mesopelagic waters of the Tara Oceans expedition to know the differences among the prokaryotic functional profiles in OMZs and non-OMZs.

## RESULTS

### Environmental characteristics.

In all sampling stations, temperature (ranging from 2.6 to 30.5°C) decreased with increasing depth (see Fig. S1a and Table S1 in the supplemental material). We found considerably lower surface water salinity in the northern (32.1 to 33.6) than in the southern (34.5 to 35.7) sampling stations (Fig. S1b). Oxygen concentrations sharply declined below the mixed layer (ca. 40- to 50-m depth) to mid-water depth (ca. 500- to 700-m depth), and the OMZ broadened from south to north (Fig. S1c). The oxygen concentration at 500 m was 6.2 to 53.2 μM (average, 24.1 μM), significantly lower (analysis of variance [ANOVA], *P* < 0.001) than that of the other three depths (surface, deep chlorophyll maximum [DCM], and 2,000 m), which ranged from 44.7 to 202.3 μM (average, 135.7 μM). Hence, we defined samples from 500 m as the BoB OMZ samples here. Among all samples, the 500-m depth of stations E87-30 and E87-32 had the lowest oxygen concentrations (6.2 μM and 7.0 μM, respectively) but the maximum nitrate concentration (46.8 μM and 39.6 μM, respectively) (Table S1). The concentration of nitrite was 0.1 to 0.4 μM in the deep samples, and the maximum concentration (0.6 μM) was detected in the DCM sample of E87-30 (Table S1). Phosphate concentration showed a similar distribution pattern as that of nitrate. Silicate concentration increased with depth (Fig. S1).

### Microbial abundance.

**FIG 1:**
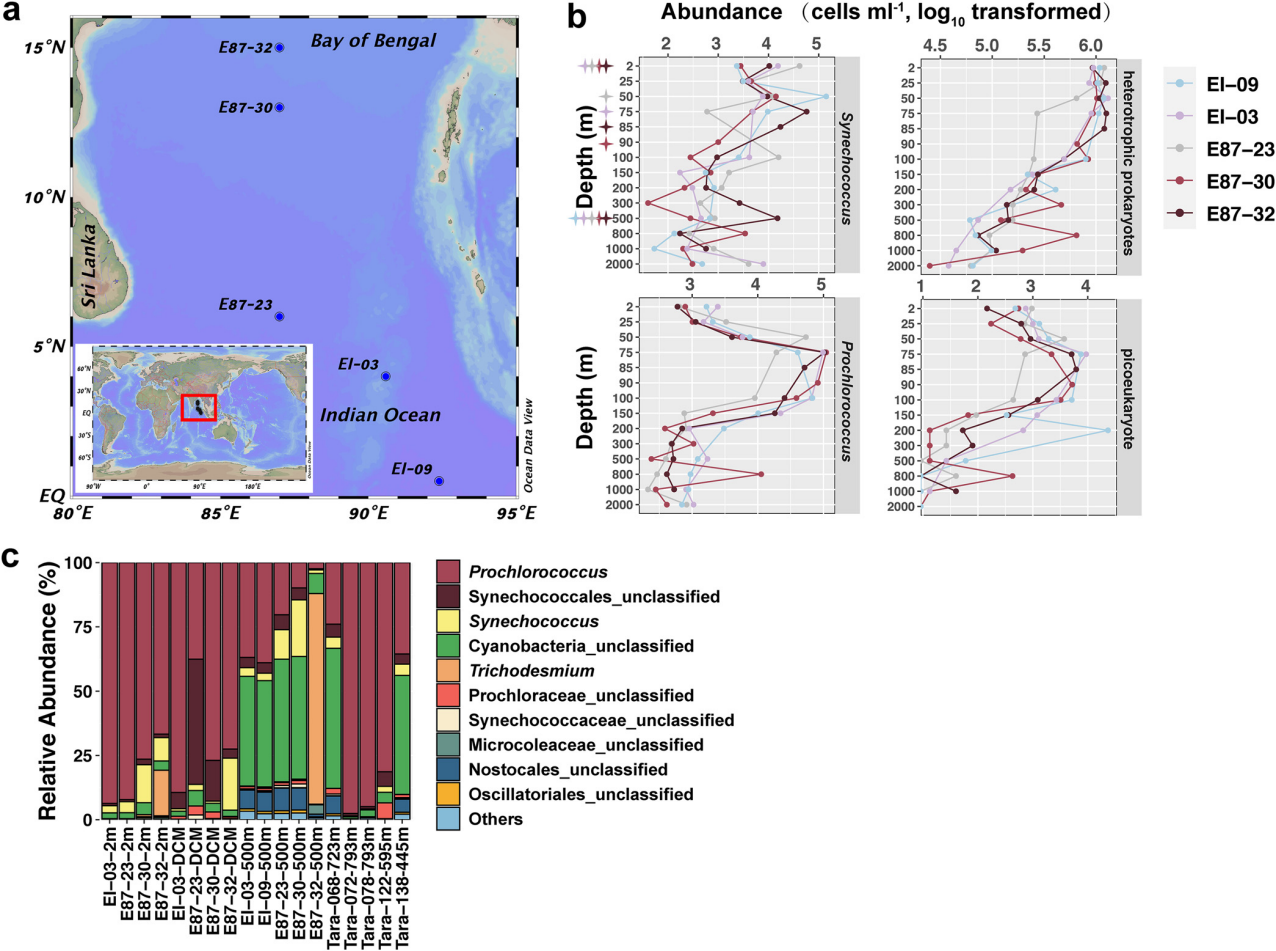
(a) Map of the study region in the Bay of Bengal showing the locations of the sampling stations. (b) Vertical distribution of microbial abundance (cells per milliliter, log_10_ transformed) including *Synechococcus*, *Prochlorococcus*, heterotrophic prokaryotes, and picoeukaryotes in the BoB. (c) Percentage of each genus in cyanobacteria (based on metagenome data). The 13 metagenomic samples of the BoB in panel c are marked in panel b at the corresponding depth with stars by different colors according to stations.

The maximum abundance (1.3 × 10^6^ cells mL^−1^) of heterotrophic prokaryotes occurred in the depth of DCM of station EI-03, while the minimum abundance (2.5 × 10^4^ cells mL^−1^) was observed at the 2,000-m depth of station E87-30 (Fig. 1b). In the middle layer (ca. 200- to 1,500-m depth), heterotrophic prokaryotes exhibited higher abundance in the north (stations E87-30 and E87-32), where oxygen concentration was relatively lower (Fig. 1b and Fig. S1). The heterotrophic prokaryotic abundances for 500-m samples of stations E87-23 (1.6 × 10^5^ cells mL^−1^), E87-30 (1.2 × 10^5^ cells mL^−1^), and E87-32 (1.4 × 10^5^ cells mL^−1^) were higher than those of stations EI-09 (6.1 × 10^4^ cells mL^−1^) and EI-03 (7.3 × 10^4^ cells mL^−1^). The highest *Prochlorococcus* abundance was observed at 75-m depth (near DCM) in stations E87-30 (1.1 × 10^5^ cells mL^−1^) and E87-32 (1.0 × 10^5^ cells mL^−1^). Below the euphotic zones (200 m), *Prochlorococcus* and *Synechococcus* can still be detected at an abundance of around 10^2^ to 10^4^ cells mL^−1^. Surprisingly, *Synechococcus* (1.5 × 10^4^ cells mL^−1^ at 500-m depth of station E87-32) and *Prochlorococcus* (1.1 × 10^4^ cells mL^−1^ at 800-m depth of station E87-30) had a relatively high abundance in the noneuphotic zone (Fig. 1b).

### Prokaryotic diversity and community composition.

The Chao1 index values of 60 samples varied from 2,177 to 4,892 based on the 16S rRNA gene analysis (Fig. S2 and Table S1). A spatial difference in the Chao1 index was observed. For example, at the DCM layer, the Chao1 index was markedly lower at stations E87-23 and E87-30 (average, 2,777.4) than at the other three stations (average, 3,958.0). The Shannon and Simpson indices were slightly higher at 500 m (average, 4.8 and 1.0, respectively) than at 2-m (average, 3.9 and 0.9, respectively), DCM (average, 4.4 and 0.9, respectively), and 2,000-m (average, 4.5 and 0.9, respectively) depths (Fig. S2). The diversity index values at each depth did not show the north-south pattern like the oxygen concentrations. Principal-coordinate analysis (PCoA) was applied to analyze the pattern of microbial community structure among the different sampled depths. Samples were generally clustered into different groups according to the sampling depth, although some samples of 2-m depth and DCM overlapped (Fig. S3). This indicated that sampling depths had distinct compositions, especially at 500-m depth, where the oxygen concentration was the lowest (Table S1). However, there was an absence of notable north-south patterns for community structure among OMZ samples.

**FIG 2:**
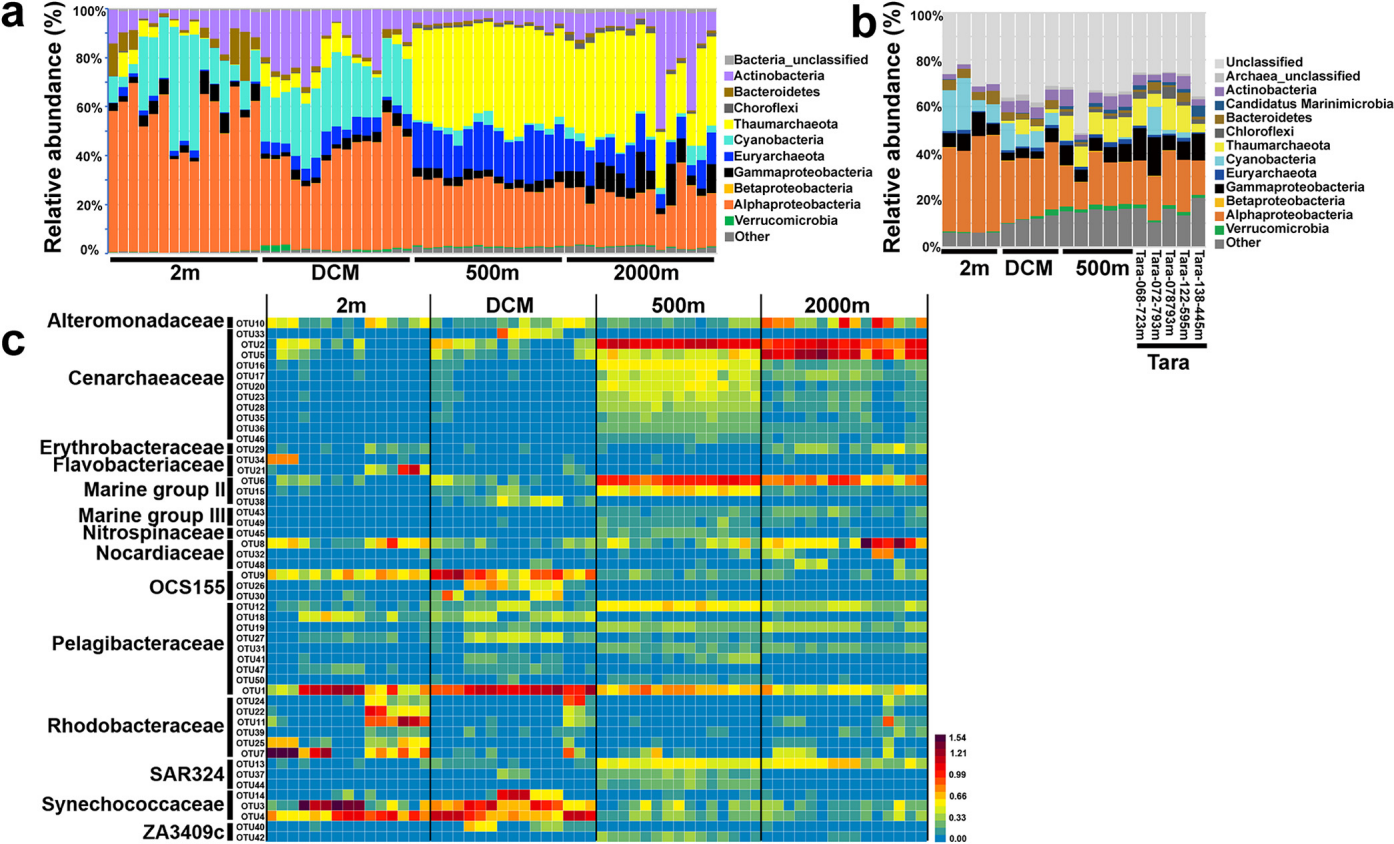
(a) Prokaryotic community composition of the BoB at the phylum or class level based on 16S rRNA gene sequences. Sequences were taxonomically classified using the *classify.seqs* command in Mothur with 80% confidence threshold, based on the SILVA version 138 reference database. The stations of each depth (*x* axis) are shown from south to north (i.e., from station EI-09 to station E87-32). Note that each sample was triplicate. (b) Prokaryotic community composition of the BoB and Tara samples at the phylum or class level based on metagenome sequences. Sequences are taxonomically classified using Diamond based on the GenBank nr database. The cutoff identified values for the class and family level are 46% and 55%, respectively, according to the work of Luo et al. (92). The stations of each depth (*x* axis) in the BoB are shown from south to north (i.e., from station EI-03 to station E87-32 for 2 m and DCM and from station EI-09 to station E87-32 for 500 m). (c) Relative abundance (log transformed) and taxonomic classification of the top 50 most abundant OTUs in the BoB.

In total, 45 bacterial phyla were identified in the 16S rRNA amplicon data sets, although most of them had a relative abundance of less than 1%. *Alphaproteobacteria*, *Cyanobacteria*, and *Actinobacteria* were predominant in the euphotic zone (2-m depth and DCM), accounting for 73.1 to 92.4% (Fig. 2a). In the deep samples (500-m and 2,000-m depth), prokaryotic communities were characterized by a higher relative abundance of *Alphaproteobacteria* (14.6 to 35.3%), *Euryarchaeota* (4.3 to 24.0%), and *Thaumarchaeota* (13.1 to 49.8%), except for the samples at 2,000-m depth of stations E87-30 and E87-32, where *Actinobacteria* (7.7 to 40.0%) were more abundant than in other samples (Fig. 2a). At the family level, samples from 2-m depth were mainly dominated by *Rhodobacteraceae*, *Synechococcaceae*, and SAR11 (*Pelagibacteraceae* in Fig. S4a), which collectively contributed to 60.4 to 89.0% of the total sequences (Fig. S4a). SAR11 (14.6 to 39.2%) was the most abundant family in DCM, followed by *Synechococcaceae* (15.8 to 29.4%) (Fig. S4a). There was an obvious shift of microbial community composition in the deep samples (500-m and 2,000-m depth), which were generally dominated by *Cenarchaeaceae* (12.7 to 52.7%) (Fig. S4a).

Prokaryotic community composition based on the 16S rRNA data sets was quite distinct from that of the metagenomes (Fig. 2b and Fig. S4). The relative abundance of archaea in the prokaryotic community compositions based on the metagenomic data set was far lower than that in the 16S-based prokaryotic community compositions at 500-m depth (Fig. 2 and Fig. S4). *Thaumarchaeota* and *Euryarchaeota* accounted for ca. 10.0% and 2.0% of the metagenomic community at the BoB 500-m depth, respectively (Fig. S4b). In station E87-32, it was worth noting that the relative abundance of *Trichodesmium* was only 17.8% in the 2-m sample but reached 81.9% in the 500-m sample, whereas the relative abundance of *Prochlorococcus* decreased from 66.7% at 2 m to 2.4% at 500 m (Fig. 1c).

Distinct distributions of the operational taxonomic units (OTUs) (based on 16S rRNA data set) within the same family were observed across the four depths (Fig. 2c). For SAR11 (*Pelagibacteraceae*), OTU1, OTU18, and OTU27 were abundant in the euphotic samples, while OTU12, OTU19, and OTU31 were more dominant in the noneuphotic zones (Fig. 2c). Although there were many *Cenarchaeaceae* members in the deep samples, they belonged to different OTUs between 500-m and 2,000-m depths. For instance, OTU2 and OTU5 were abundant at both 500 m and 2,000 m, whereas OTU16, OTU17, OTU20, OTU23, OTU28, OTU35, OTU36, and OTU46 had a higher abundance at the 500-m than at the 2,000-m depth (Fig. 2c).

### Prokaryotic network structure complexity and stability.

The network of prokaryotic communities (based on 16S rRNA data set) at each depth demonstrated distinct cooccurrence patterns (Fig. S5 and Table S2). A network containing 284 nodes and 1,357 links was obtained for prokaryotic communities at 2-m depth, and the network was fragmented into 9 modules (Fig. S5a and Table S2). The prokaryotic communities of DCM resulted in a network of 317 nodes and 1,153 links that was fragmented into 19 modules (Fig. S5b and Table S2). Significantly, the network of the 500-m depth was constructed by 219 nodes and 156 links, and the network was fragmented into 72 modules (Fig. S5c and Table S2). A network of 336 nodes and 575 links was obtained for prokaryotic communities at 2,000-m depth, and the network was fragmented into 59 modules (Fig. S5d and Table S2). Values of average degree and average clustering coefficient for 500-m networks were the lowest (Table S2). A markedly higher modularity and number of modules in the OMZ samples (1.0 and 72, respectively) than in other layers (Fig. S5 and Table S2) were observed (Fig. S5c and d and Table S2), which suggests the simplicity and instability of the OMZ network structure in the BoB.

In the 2-m-depth and DCM networks, nodes were mainly comprised of *Rhodobacteraceae* (34.2%) and SAR11 (18.0%), respectively. Nevertheless, at 500-m and 2,000-m depths, the nodes mainly belonged to the archaeal family *Cenarchaeaceae* (23.7% and 18.5%, respectively) (Fig. S5). SAR11 also accounted for a significant proportion of nodes at 2-m (14.8%), 500-m (18.7%), and 2,000-m depths (9.5%) (Fig. S5). *Synechococcaceae*, which had a high relative abundance of the community composition in the euphotic zones, contributed only 6.7% and 5.4% of the nodes in 2-m-depth and DCM networks, respectively, but they were associated with a high number of other OTUs (e.g., SAR11 and *Alteromonadaceae*) in the networks (Fig. S5).

### Relationship between prokaryotic community composition and environmental variables.

Redundancy analysis (RDA) was performed to identify possible linkages between the prokaryotic community composition and environmental variables. Nitrate, depth, dissolved oxygen (DO), and salinity were the most important factors for the community differences between the euphotic zone (2-m depth and DCM) and deep samples (500-m and 2,000-m depth) (Fig. S6a). At the euphotic zone, temperature and nitrite were crucial variables determining the prokaryotic community composition (Fig. S6b). In contrast, DO and P played a vital role in the community differences in the deep samples (Fig. S6c).

Linear regression analyses showed that the relative abundance of all dominant archaeal groups, including *Cenarchaeaceae*, Marine Group II, and Marine Group III, exhibited significant negative correlation with DO concentration (*R*^2^ = 0.38, *P* < 0.001; *R*^2^ = 0.67, *P* < 0.001; and *R*^2^ = 0.31, *P* < 0.001, respectively) (Fig. S7). However, the relative abundances of the most dominant bacterial families (e.g., *Synechococcaceae* and *Rhodobacteraceae*) were positively correlated with oxygen concentration (Fig. S7).

### Metagenomic analysis of nitrogen metabolism.

**FIG 3:**
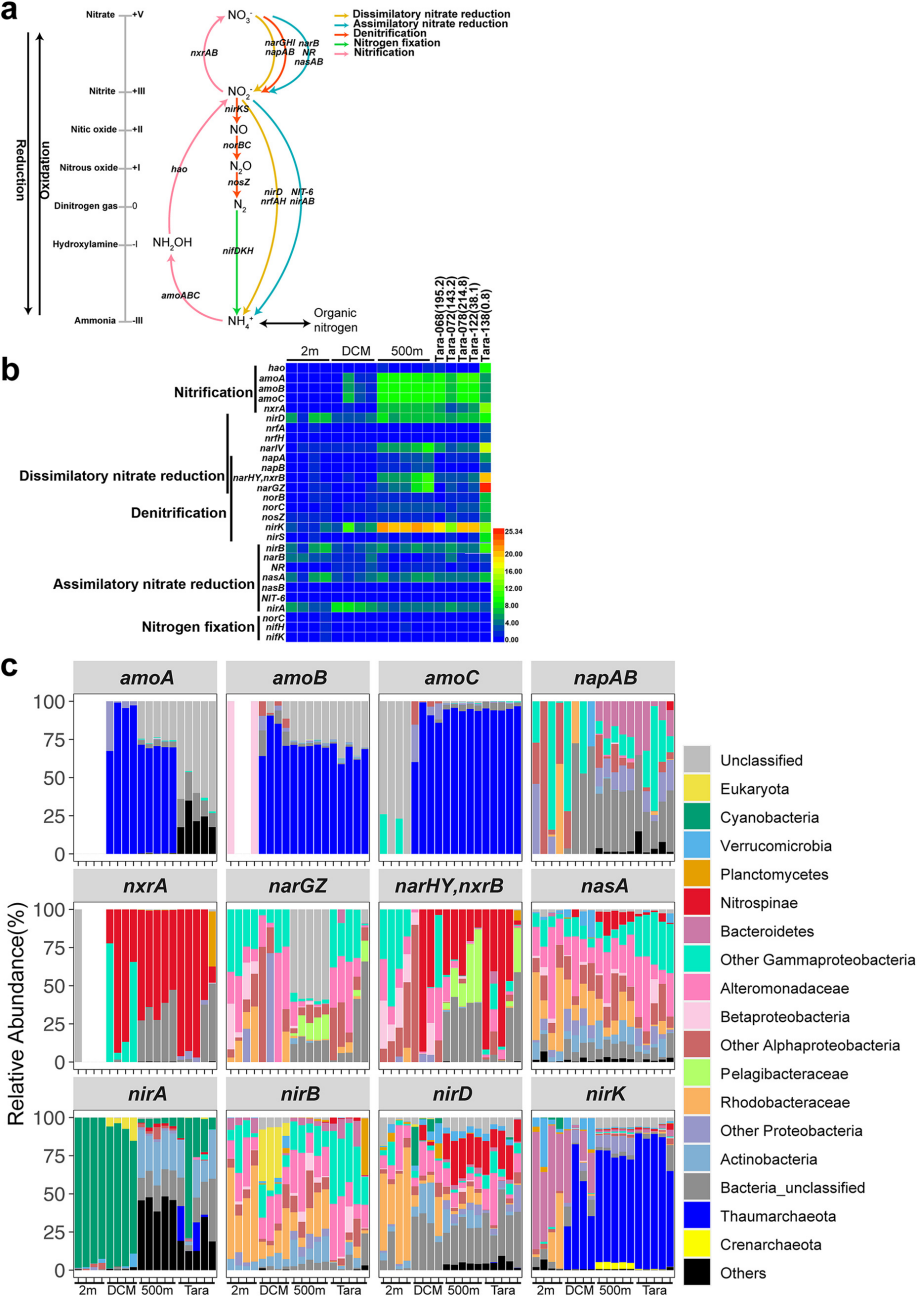
(a) Nitrogen cycling potential in the BoB. (b) TPM value (square transformed) of nitrogen metabolism-related genes. The stations of each depth (*x* axis) in the BoB are shown from south to north (i.e., from station EI-03 to station E87-32 for 2 m and DCM and from station EI-09 to station E87-32 for 500 m). Oxygen concentrations (micromolar) of Tara samples are shown in the parentheses after sample names. (c) Microbial taxa of the genes and their relative abundance in each sample. The stations of each depth (*x* axis) in the BoB are shown from south to north (i.e., from station EI-03 to station E87-32 for 2 m and DCM and from station EI-09 to station E87-32 for 500 m), and the Tara samples are shown in the same order as in panel b.

The genes of nitrogen metabolism in the BoB were mainly involved in pathways of assimilatory nitrate reduction (*narBGHI*), denitrification (*nirK*, *norBC*, and *nosZ*), nitrogen fixation (*nifKH*), and nitrification (*nxrAB* and *amoABC*) (Fig. 3a). In general, the *amoABC* (ammonium monooxygenase), *narGH* (nitrate reductase), and *nirK* (nitrite reductase) genes were more abundant in OMZ samples than in other water layers (Fig. 3 and Table S3), indicating potentially more active ammonia oxidation, nitrate reduction, and denitrification in the BoB OMZ than in the other depths. The *narGH* genes were more abundant at E87-30-500 m, E87-32-500 m, and Tara-138, where oxygen concentration was the lowest (Fig. 3b and Table S1). The values of transcripts per million (TPM) of SAR11 *narGH* genes (nitrate reductase) markedly increased at E87-30-500 m, E87-32-500 m, and Tara-138 (Fig. 3c) and showed a close relationship with oxygen concentration (see Fig. 5a and b, linear model analysis, *R*^2^ = 0.78 and 0.75, respectively). This indicates the potential importance of SAR11 in anaerobic nitrate reduction in the BoB. The taxonomy of *amoABC* and *nirK* genes found at the DCM and 500-m depth of the BoB and Tara samples mainly belonged to *Thaumarchaeota* (Fig. 3c). The *nirA* gene (belonging to assimilatory nitrite reduction), which transforms nitrite to ammonium, was mostly contributed by *Cyanobacteria* at 5-m depth and DCM but mainly by *Actinobacteria* at 500-m depth (Fig. 3c). In addition, a relatively high abundance of the *nxrA* gene affiliated with *Nitrospinae* was observed at 500-m depth (Fig. 3c).

### Metagenomic analysis of sulfur metabolism.

**FIG 4:**
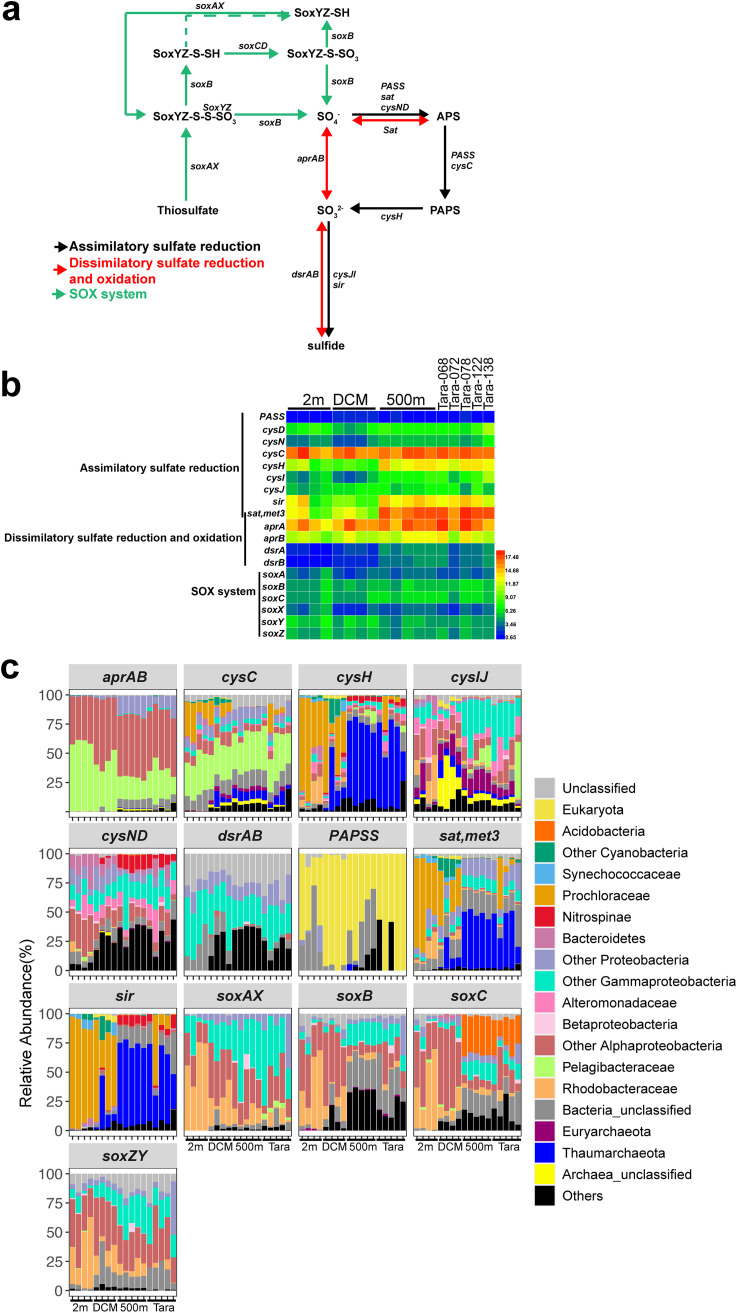
(a) Sulfur cycling potential in the BoB. APS, ammonium persulfate; PAPS, 3′-phosphoadenylyl sulfate. (b) Absolute abundance (square transformed) of sulfur metabolism-related genes. (c) Microbial taxa of the genes and their relative abundance in each sample. For panels b and c, the stations of each depth (*x* axis of panels b and c) in the BoB are shown from south to north (i.e., from station EI-03 to station E87-32 for 2 m and DCM and from station EI-09 to station E87-32 for 500 m), and the Tara samples are shown in the same order as in Fig. 3b.

**FIG 5:**
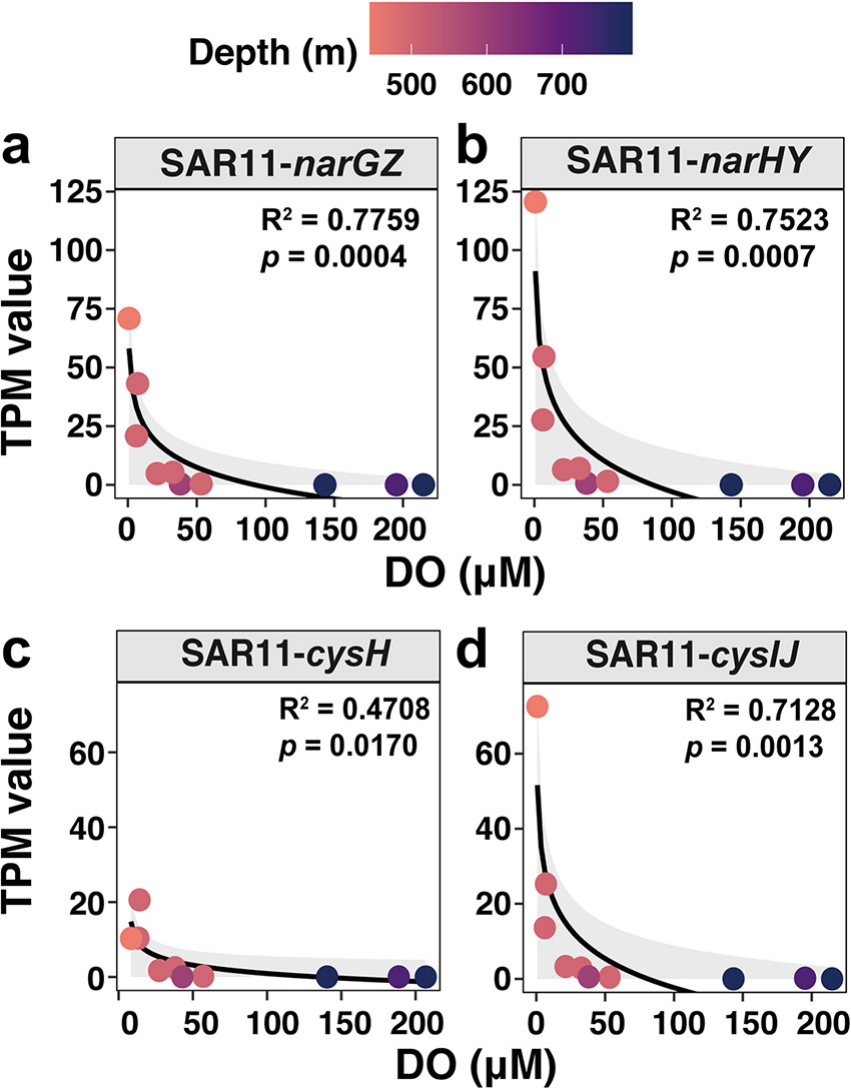
The linear model between DO concentration and TPM value of the *nar* gene (a and b) and *cys* gene (c and d) affiliated with SAR11 (*Pelagibacteraceae*) at 500-m samples of the BoB and Tara samples. The gray shadow indicates the confidence interval.

The sulfur cycle genes were predominated by assimilatory sulfate reduction (*cysCDHNIJ*), dissimilatory sulfate reduction and oxidation (*aprAB* and *dsrAB*), and sulfur-oxidation (SOX) system (*soxABCXYZ*) genes (Fig. 4a). These results showed that genes related to the sulfur cycle were abundant in the BoB, especially in the 500-m layer compared to euphotic zones (Fig. 4b). The *cysI* (sulfite reductase), *sir* (sulfite reductase), and *dsrAB* (sulfur oxygenase and sulfate reductase) genes were more abundant at 500-m depth than at the 2-m depth and DCM of the BoB (Fig. 4b and Table S4). These genes were contributed by distinct microbial taxa in the euphotic zone (2-m depth and DCM) and the deep ocean (500-m depth and Tara) (Fig. 4c). For example, *cysH* (3′-phosphoadenosine 5′-phosphosulfate reductase), *sat* (sulfate adenylyl transferase), and *sir* primarily belonged to *Prochloraceae* at 2-m depth and DCM of the BoB, whereas they predominantly belonged to *Thaumarchaeota* at 500-m depth of the BoB and Tara samples (Fig. 4c). It was notable that the relative contributions by SAR11 to the *cysHIJ* markedly increased at E87-30-500 m, E87-32-500 m, and Tara-138, where oxygen concentration was the lowest (Fig. 4c and Table S1). Furthermore, the linear model showed a significant correlation (*R*^2^ = 0.47 and 0.71, respectively) between oxygen concentration and TPM values of SAR11 *cys* genes (Fig. 5c and d), suggesting the importance of SAR11 in the BoB OMZ.

## DISCUSSION

Overall, we detected an extensive OMZ in the BoB, where prokaryotes perform diverse metabolisms under hypoxic conditions. The lowest oxygen concentration (6.21 μM) among the four sampling depths of the five stations was higher than previously described in the BoB OMZ (below the detection limit of 7 to 12 nM and 0.6 μM, respectively) (20, 27). It was also higher than that detected in other OMZs, such as AS, ETNP, and ETSP (below the detection limit) (3, 4, 29, 30). Hence, the present study expands the current knowledge about prokaryotic diversity and its potential function in the OMZs where oxygen concentration is still above the suboxic level.

### The sinking of *Trichodesmium* in the BoB.

The cyanobacteria *Synechococcus* and *Prochlorococcus* are always detected with a high abundance in the euphotic zones above the OMZs (31–33), and their sinking provides a considerable carbon source for OMZs (34). For example, the abundance of *Synechococcus* and *Prochlorococcus* in the OMZ of the Costa Rica Dome peaked at 10^6^ cells mL^−1^ (35). Similarly, a high *Synechococcus* and *Prochlorococcus* abundance (up to 10^5^ cells mL^−1^) was observed in the BoB euphotic zones (Fig. 1b). Furthermore, the metagenomic data showed a dominance of *Prochlorococcus* among the cyanobacterial community in the BoB euphotic zones (Fig. 1c). Also, up to 1.13 × 10^4^ cells mL^−1^ of *Prochlorococcus* were detected at 800-m depth. They could be from the sinking of upper layers or could survive in the deep ocean waters (36, 37). At station E87-32, we observed a *Trichodesmium* bloom on the sea surface during the sampling. The metagenomic analysis estimated that *Trichodesmium* constituted 17.76% of the cyanobacterial community (Fig. 1c). The *Trichodesmium* bloom at the surface water of E87-32 was not reflected in the 16S rRNA amplicon and metagenomic data set at the DCM of the same station. The DCM layer is predominated by other cyanobacteria like *Prochlorococcus* and *Synechococcus* that contribute the majority of the sequence result. Interestingly, despite the fact that *Trichodesmium* was not the most dominant cyanobacterium in the surface layer (17.6%) of the station E87-32, its relative abundance increased to 81.9% at 500 m (Fig. 1c), suggesting that the sinking of *Trichodesmium* was far more efficient than that of other cyanobacteria (e.g., *Prochlorococcus*) into the OMZ layer. This was possibly caused by the increase of transparent exopolymers by programmed cell death of *Trichodesmium*, which can happen due to phosphorus starvation, resulting in a massive downward pulse of particulate organic matter (29, 30). *Trichodesmium* is known as a nitrogen fixer and a source of dissolved organic nitrogen/ammonium (38). In the present study, the ammonium concentrations in the whole water column of station E87-32 were higher (average ca. 2- to 4-fold) than those in other water columns and the high concentration continued even to 2,000-m depth (see Fig. S1g in the supplemental material). Given the abundant *Trichodesmium* population inhabiting surface waters of the BoB throughout the year (21, 39, 40), it is reasonable to speculate that *Trichodesmium* has a considerable contribution of carbon and nitrogen input into the OMZ layer and even the deeper ocean for the maintenance of the BoB OMZ. However, we found a low abundance of nitrogen fixation-related genes (*nif*) in the surface layer of station E87-32 and no *nif* genes in the OMZ samples of the BoB. This was different from the study in the OMZ off Peru, where nitrogen fixation within OMZ waters was detected (41), but was very similar to work in offshore anoxic OMZs (42–44). Diazotrophic activities may frequently be relevant to organic carbon availability (41, 42, 44), and it partially explains the different distributions of *nif* genes between coastal and offshore OMZs.

### Specific prokaryotic community of the BoB.

Decomposition of sinking organic materials could result in the decrease of oxygen concentration, which would restructure the prokaryotic community in the OMZ. In the present study, euphotic zone communities were dominated by *Alphaproteobacteria* and *Cyanobacteria*, while the community composition shifted toward *Thaumarchaeota* and *Euryarchaeota* in deeper waters (500- and 2,000-m depth) (Fig. 2a and b). The *Synechococcaceae* made a relatively higher contribution at 2-m depth of stations EI-03 and E87-23 (Fig. S4a) than in 2-m-depth samples of other stations. The PCoA further indicated the prokaryotic community of these two samples was more closely related to the prokaryotic community at the DCM (Fig. S3). Consistently, a peak in *Synechococcus* abundance was observed in these two sample types (Fig. 1b). This pattern was possibly caused by the upwelling in the equatorial eastern Indian Ocean, which is predominantly forced by atmospheric intraseasonal oscillations and shows larger amplitudes during spring (45) when the sampling was conducted (April-May). Therefore, nutrients were brought to the surface water to support the *Synechococcus* growth.

Below the euphotic zones, *Euryarchaeota* and *Thaumarchaeota* accounted for 4.3 to 24.0% and 13.1 to 49.8% of total prokaryotic communities, respectively. Such a high relative abundance of archaea was not observed in previous studies in OMZs, no matter whether they used the same primers (46) or different primers (26, 27) from this study. For example, Pajares et al. (46) found *Euryarchaeota* and *Thaumarchaeota* accounted for only <5% and <25% of the prokaryotic communities at 500-m depth of the ETNP, respectively, where these communities were dominated by *Alphaproteobacteria* (ca. 50%). However, such a high relative abundance of archaea was not found in the metagenomic data (Fig. 2b and Fig. S4b), which is possibly due to the primer bias. Parada et al. (47) found the primer 806R underestimated SAR11 taxa, and they observed an increase in *Thaumarchaea* coverage when using 806R compared to 926R. Therefore, for the 16S data of this study, the primer (341F/806R) may result in underestimation of the abundance for SAR11 and the overestimation of that for *Thaumarchaeota*. Another alternative explanation is that the number of archaea in the metagenome was underestimated given the fact that a large proportion of reads (32.7% to 35.7%) were “unclassified” and that the archaea are generally more poorly represented in databases than bacteria.

PCoA showed a clear difference between samples from 2-m, DCM, 500-m, and 2,000-m depths (Fig. S3). Furthermore, an independent network was observed at the 500-m depth of the BoB compared with other depths (Fig. S5). These implied a special prokaryotic community structure and a less stable prokaryotic network in the BoB OMZ. Similar studies in OMZs observed that microbial communities from the OMZ form distinct clusters from the euphotic zone and the deep ocean (22, 24, 48). The specificity of the prokaryotic community would result from the unique physical and chemical factors (e.g., temperature, salinity, and DO) of OMZs (1, 49). At 500-m depth, the prokaryotic community was negatively correlated with temperature, salinity, and DO, similar to the findings of Fernandes et al. (27) in the BoB OMZs. These factors were reported as major factors influencing prokaryotic community composition (27, 50).

### Nitrogen metabolism in the BoB OMZ.

As aerobic and anaerobic microbial processes operate at the same time in waters with low or even undetectable oxygen concentrations, characterizing the response of microbial community functions to oxygen fluctuation can be complex (49). For example, a recent model predicted 0.1 to 10.0 nM as the minimum oxygen concentration for maintaining aerobic metabolism in OMZs (51). Also, actual data measured by shipboard incubations showed there were negligible rates of ammonia oxidation and high rates of nitrite oxidation at anoxic depths of the ETNP and ETSP (52–54), while both processes were detected in the OMZ off Chile at low-nanomolar oxygen levels (55). In this study, the *amoABC* genes were highly abundant in OMZ samples of the BoB compared to anoxic Tara samples (Tara-138) but similarly abundant as in oxic Tara samples (Fig. 3b), indicating potentially enriched nitrification in the BoB OMZ. Prokaryotic taxa involved in these processes were mainly *Thaumarchaeota*. Also, about 70% of detected *nirK* reads belonged to *Thaumarchaeota* (Fig. 3c), which are known to contain multiple copies of *nirK*-like copper oxidase-encoding genes (56). In the BoB OMZ, the dominance of archaeal over bacterial ammonia oxidizers was observed (Fig. 3c), which is similar to findings in other OMZs (25, 52, 57, 58) and oxic ocean waters (59, 60). Given the high N_2_O production rates by archaeal ammonia-oxidizers under low oxygen concentrations (61), the production of this greenhouse gas from OMZs would be enhanced by OMZs expanding.

SAR11 bacteria are often the most abundant prokaryotes in the OMZs, as observed in a number of studies (10, 22, 25) as well as this study (Fig. S4). SAR11 has an important role in encoding proteins catalyzing the reduction of nitrate to nitrite in OMZs (10). In this study, it was surprising that the SAR11-*nar* genes were detected even in the incompletely anoxic BoB (Fig. 3c). Then, we observed a significant negative linear relationship between oxygen concentration and the TPM value of the SAR11-*nar* gene (Fig. 5a and b), suggesting the potential importance of SAR11 in nitrate reduction in the BoB OMZ. This result also indicates that there will be more SAR11 bacteria to use nitrate after the BoB expands and goes anoxic.

However, the nitrite-N in the OMZ of the BoB seemed to be not further reduced to nitrogen gas. The quite low abundance of *norBC* and *nosZ* indicates the N_2_ loss in the BoB was limited compared to that in other OMZs (20). The oxygen concentration of the BoB ranged from 6 to 200 nM, which would allow nitrite oxidation by *Nitrospinae* (20). The relatively high abundance of the *nxrA* gene affiliated with *Nitrospinae* suggests this family as the major nitrite-oxidizing bacteria (NOB) in the BoB OMZs as observed by other studies (62). Therefore, the nitrite available for denitrification in the BoB was possibly restricted (20) due to the competition between NOB and denitrifiers for nitrite. Furthermore, the abundance of the *nirK* gene in low-hypoxic BOB samples was similar to those in high-hypoxic BoB and oxic Tara samples, and most of the *nirK* genes detected in BoB OMZ samples were affiliated with *Thaumarchaeota* (Fig. 3b and c). Previous studies have shown that the *nirK* gene affiliated with *Thaumarchaeota* is proposed to be involved in archaeal ammonia oxidation (63, 64) but not nitrite reduction. These explained that although the abundance of the denitrification-related genes (*nar* and *nirK*) was relatively high in the BoB OMZ, the actual N_2_ loss from the BoB is generally low compared to that from other OMZs (20). However, if the oxygen level of BoB OMZ continues to decline due to warming (65, 66), nitrite oxidation will be suppressed and nitrite will accumulate (20, 67). This may lead to the enhancement of denitrification/anammox and therefore may increase N_2_ loss in the BoB OMZ in the future.

### Summary.

In this study, we showed the prokaryotic community and its potential functions in nitrogen metabolism in the BoB OMZ, a pronounced oxygen minimum zone. We highlighted the importance of *Trichodesmium* as a carbon and nitrogen source in the BoB OMZ. Additionally, a large number of archaeal-*amo* (primarily affiliated with *Thaumarchaeota*) and SAR11-*nar* genes were detected in the low-hypoxic (>5 to ≤20 μM O_2_) BoB waters. This scene will be more widespread after suboxic waters expand. Higher proportions of *nar* genes were affiliated with SAR11 as the oxygen concentrations decreased, indicating that more SAR11 bacteria will use nitrate in the future ocean if the oxygen levels decrease. Nitrite-N so far was not further reduced to nitrogen through denitrification but likely oxidized to nitrate by *Nitrospinae* in the BoB OMZ. However, if the BoB OMZ becomes anoxic, the lack of nitrogen production will change.

## MATERIALS AND METHODS

### Sample collection and determination of environmental parameters.

Cruises onboard the R/V *Shiyan 3* were carried out in the Eastern Indian Ocean from 22 March to 12 May 2019 (Fig. 1a). Seawater was collected at 14 depths (2, 25, 50, 75, 85, 90, 100, 150, 200, 300, 500, 800, 1,000, and 2,000 m) at stations EI-09, EI-03, E87-23, E87-30, and E87-32 (Fig. 1a) using 8-L Niskin bottles assembled on a rosette with conductivity-temperature-depth (CTD, Seabird SBE-911) mounted with a calibrated oxygen sensor (Seabird SBE-43). The DCM in each sampling station was varied from 50 m to 90 m (see Table S1 in the supplemental material). For picoplankton (heterotrophic prokaryotes, *Synechococcus*, *Prochlorococcus*, and picoeukaryotes) abundance measurement, 2 mL seawater was prefiltered by 20-μm mesh and fixed with formaldehyde (with a final concentration of 1.5%). For prokaryotic community (samples at 2-m, DCM, 500-m, and 2,000-m depth) and metagenomic (samples at 2-m, DCM, and 500-m depth) analysis, 10 L prefiltered seawater was filtered onto 0.2-μm (47-mm-diameter) polycarbonate membranes (Millipore) by an aspirator. Samples were stored at −80°C until analysis. For the measurement of inorganic nutrients (ammonia, nitrate, nitrite, phosphate, and silicate), seawater was filtered through a 0.7-μm-pore-size glass fiber filter (Whatman) and stored in the 80-mL polyethylene bottles at −20°C.

In the laboratory, the inorganic nutrients were measured by a flow-injection autoanalyzer (Quickchem 8500; Lachat Instruments, USA) according to the classical colorimetric methods (68). For determination of chlorophyll *a* (Chl *a*), 500 mL of seawater was filtered through a 0.7-μm-pore-size GF/F filter (Whatman) and then stored at −20°C. Chl *a* was extracted with 90% (vol/vol) acetone and measured using a Turner Design 10-AU fluorometer (69).

### Picoplankton abundance.

Three picophytoplankton populations (*Prochlorococcus*, *Synechococcus*, and picoeukaryotes) were enumerated using a Becton Dickinson FACSCalibur flow cytometer based on side light scatter (SSC) and orange and red fluorescence parameters according to a previous protocol (70). To measure heterotrophic prokaryotic abundance, a portion of the fixed samples were stained with the nucleic acid dye SYBR green I (Sigma-Aldrich Co., USA) (final dilution 10^−4^, vol/vol), followed by dark incubation for half an hour. Heterotrophic prokaryotic abundance was detected based on SSC and green fluorescence (FL1, 530- ± 15-nm) signals.

### DNA extraction, PCR, and pyrosequencing.

Genomic DNA was extracted from the 0.2-μm membranes using the modified enzyme/phenol-chloroform extraction protocol (71, 72). In brief, each membrane was cut into small pieces and then transferred into a 2-mL tube with 0.5 mL of solution I (50 mM EDTA, 50 mM Tris-HCl, and 50 mM sucrose; pH 8.0). Lysozyme (5 mg mL^−1^, final concentration) was added into the tubes after three freezing-and-thaw cycles using liquid nitrogen and a 60°C dry bath and then incubated for 1 h at 37°C. Proteinase K (2 mg mL^−1^, final concentration) together with sodium dodecyl sulfate (0.5%, wt/vol) was added and further incubated for 2 h at 60°C. Then, DNA was extracted from each sample with an equal volume of phenol-chloroform-isoamyl alcohol (25:24:1). After being centrifuged for 10 min at 12,000 × *g*, the upper aqueous layer was transferred to a fresh microcentrifuge tube. Then, samples were extracted twice with an equal volume of chloroform-isoamyl alcohol (24:1). The supernatants were transferred to fresh microcentrifuge tubes, and isopropyl alcohol (i.e., 70% volume of the supernatant) was added. After incubation at −20°C overnight, the DNA in each tube was concentrated by centrifugation at 12,000 × *g* for 10 min. The DNA pellets were washed twice using 0.2 mL 70% ethanol and resuspended in 35 μL TE buffer (1 mM EDTA, 10 mM Tris-HCl; pH 8.0).

The V3 and V4 regions of the 16S rRNA gene were amplified with modified primers 341F (5′-CCTAYGGGRBGCASCAG-3′) and 806R (5′-GGACTACNNGGGTATCTAAT-3′) (73) for pyrosequencing. The primers have previously been reported to target both bacteria and archaea (73). The PCR was carried out in a 25-μL master mix, including 1 μL of DNA, 0.5 μM (each) primer, 1.5 mM MgCl_2_, 0.2 mM (each) deoxynucleoside triphosphate (dNTP), 1× PCR buffer, and 1.0 unit of Platinum *Taq* DNA polymerase (Invitrogen). We used sterilized water as the negative control. The PCR for each sample was carried out in triplicate with the following thermal cycles: 5-min initial denaturation at 95°C, followed by 30 cycles of 95°C for 30 s, 55°C for 30 s, and 72°C for 60 s, followed by a final extension at 72°C for 7 min before holding at 4°C. The paired-end amplicon sequencing was conducted by the Magigene Company (China) using the Illumina Hiseq 2500 platform.

The metagenome sequencing was conducted by the Novogene Company (China). Briefly, a total amount of 1 μg DNA per sample was used as input material for the DNA sample preparations. Sequencing libraries were generated using the NEBNext Ultra DNA library prep kit for Illumina (NEB, USA) following the manufacturer’s recommendations, and index codes were added to attribute sequences to each sample. The clustering of the index-coded samples was performed on a cBot cluster generation system according to the manufacturer’s instructions. After cluster generation, the library preparations were sequenced on an Illumina PE150 platform, and paired-end reads were generated.

### Analysis of 16S rRNA gene amplicons.

Amplicons were analyzed with the software Mothur, according to the standard protocol (74). In brief, tags and primers were trimmed first using the command *trim.seqs*. Sequences with an average quality score below 20 and lengths shorter than 300 bp were removed, and then all sequences were aligned against the SILVA version 138 reference database (75) using the command *align.seqs*. The *filter.seqs* command was used to remove columns where every character is either a “.” or a “-.” The *chimera.uchime* command was used to analyze and remove chimeras. Then, the high-quality sequences were identified with the Greengenes database version 13.8 at a cutoff value of 60% (76). Sequences identified as chloroplasts, mitochondria, or unknown were removed, and remaining sequences were clustered into operational taxonomic units (OTUs) with cutoff values of 3%. The *remove.rare* command was used to removed singletons (OTUs with just one sequence). To rarefy the data sets to an equal sequencing depth, 20,000 sequences were subsampled from each sample for subsequent analysis. This number of sequences subsampled was also due to the limitation of our computer capability. The *classify.otu* command was used to identify OTUs against the Greengenes version 13.8. database (76). Subsequently, the *rarefaction.single* command was used to analyze the alpha diversity of the samples.

### Analysis of metagenomes.

Metagenomic analysis was conducted using the SqueezeMeta pipeline (77). Five metagenomic samples from the Tara Oceans data set were downloaded from the ENA database (http://www.ebi.ac.uk/ena/data/view/) and also analyzed (Table S1). Metagenomic reads were quality checked and trimmed for low-quality regions using Trimmomatic (78). Then, sequences were assembled using Megahit with default settings (79). Open reading frames (ORFs) of the assembled contigs (>200 bp) which were identified by the Prodigal software (80) were further annotated using DIAMOND against both the NR and KEGG databases, with an E value cutoff of 1 × 10^−3^ (81). Raw reads were mapped to the contigs using Bowtie to calculate the abundance of each ORF (82). The abundance of each ORF was calculated as TPM = rg × rl × 10^6^/cl × *T*, where rg is reads mapped to gene *g*, rl is read length, cl is coding sequence (CDS) length, and *T* is the sum of rg × rl/cl for all genes (83). Because *narH*, *narY*, and *nxrB* genes are closely related to each other and annotated with the same KEGG orthology (K00371), their abundances were calculated together.

### Statistical analysis.

Samples were categorized into different oxygen regimes. We defined the hypoxic (>5 to ≤60 μM O_2_) as one regime including the low-hypoxic (>5 to ≤20 μM O_2_) and high-hypoxic (>20 to ≤60 μM O_2_) regimes, representing important thresholds of oxygen concentrations for biological processes (84). Oxygen concentrations of >60 μM were defined as “oxic.”

The map of sampling stations and vertical distributions of environmental parameters were displayed by Ocean Data View version 5.2.0 (85). All heatmaps including the relative abundance of the 50 most abundant OTUs (log-transformed) and metabolism-related gene abundance (square root transformed) were generated by HemI (86). The ordination of the prokaryotic community visualized by a PCoA plot and RDA evaluating the relationships between prokaryotic community and environmental variables were done using the “vegan” package version 2.5-7 in R version 4.0.3 (87, 88). To understand the cooccurrence network structures among the prokaryotic OTUs at different depths (2 m, DCM, 500 m, and 2,000 m), we employed a molecular ecological network (MEN) analysis (89). Network calculations were based on Spearman rank correlation coefficients with a cutoff of 0.80, and the networks were visualized by Cytoscape version 3.7.2 (90). We used the network topological parameters of nodes, edge numbers, average degree, modularity, and module to assess prokaryotic network complexity and stability. Higher node, higher edge numbers, higher average degree, and smaller modules represent greater network complexity, indicating higher stability of the ecosystem (91). To test the differences of abundance for metabolic genes between different depths, analysis of variance (ANOVA) was conducted by SPSS version R26.0. The “lm” function in R was used to analyze the correlation between TPM value of selected genes and oxygen concentration with the linear regression model.

### Data availability.

All sequences obtained from this study have been deposited in the National Center for Biotechnology Information (NCBI) Sequence Read Archive under accession number PRJNA724894. The CTD data of the five stations in this study in the BoB have been deposited at the website http://data.scsio.ac.cn/metaData-detail/1503343885621309440.
