# Supplementary material for: Insights into Prokaryotic Community and Its Potential Functions in Nitrogen Metabolism in the Bay of Bengal, a Pronounced Oxygen Minimum Zone
Source: Microbiol Spectr. 2022 May 17;10(3):e00892-21. doi: 10.1128/spectrum.00892-21 (PMC9241787; doi:10.1128/spectrum.00892-21)
Supplement: SUPPLEMENTAL FILE 1 — Supplemental material. Download spectrum.00892-21-s0001.pdf, PDF file, 1.6 MB [file spectrum.00892-21-s0001.pdf]

# Insights into prokaryotic community and its potential functions in nitrogen metabolism in the Bay of Bengal, a pronounced oxygen minimum zone

**Bowei Gu,[a,b,c#] Jiaxing Liu,[a,b,c#] Shunyan Cheung,[d,e] Ngai Hei Ernest Ho,[d] Yehui Tan,[a,b,c] Xiaomin Xia[a,b,c*]**

[a] Key Laboratory of Tropical Marine Bio-resources and Ecology, South China Sea Institute of Oceanology, Chinese Academy of Sciences, Guangzhou, China

[b] University of Chinese Academy of Science, Beijing, China

[c] Southern Marine Science and Engineering Guangdong Laboratory (Guangzhou), Guangzhou, China

[d] Department of Ocean Science, The Hong Kong University of Science and Technology, Hong Kong, China

[e] Hong Kong Branch of Southern Marine Science and Engineering Guangdong Laboratory, The Hong Kong University of Science and Technology, Hong Kong, China

*** Correspondence:**

Xiaomin Xia

xxia@connect.ust.hk

# B.G. and J. L. contributed equally to this work.

The supplementary information includes:

- 4 tables (Table S1 to S4)
- 7 figures (Fig. S1 to S7)

**Table S1.** Environmental parameters of different depths at sampling sites and the Tara samples.

| Station | Longitude (ºE) | Latitude (ºN) | Depth (m) | Temperature (℃) | Salinity | DO (μM) | $NH_4^+$ (μM) | $NO_2^-$ (μM) | $NO_3^-$ (μM) | $PO_4^-$ (μM) | $SiO_3^-$ (μM) | Chl *a* (μg/L) | 16S rRNA | Metagenomics |
|---|---|---|---|---|---|---|---|---|---|---|---|---|---|---|
| EI-09 | 92.4 | 0.5 | 2 | 30.4 | 35.7 | 202.1 | 0.8 | 0.3 | 1.2 | 0.1 | 2.6 | 0.1 | √ | |
| | 92.4 | 0.5 | 75 (DCM) | 29.1 | 34.6 | 185.0 | 1.1 | 0.3 | 1.4 | 0.1 | 3.2 | 0.6 | √ | |
| | 92.4 | 0.5 | 500 | 9.8 | 35.0 | 53.2 | 2.6 | 0.4 | 35.4 | 2.5 | 57.1 | 0.0 | √ | √ |
| | 92.4 | 0.5 | 2000 | 2.7 | 34.8 | 130.3 | 2.4 | 0.4 | 38.3 | 2.8 | 165.4 | 0.0 | √ | |
| EI-03 | 90.6 | 4.0 | 2 | 30.2 | 34.5 | 156.3 | 1.7 | 0.3 | 1.3 | 0.1 | 2.2 | 0.1 | √ | √ |
| | 90.6 | 4.0 | 75 (DCM) | 28.1 | 35.1 | 172.2 | 0.9 | 0.4 | 2.1 | 0.2 | 3.2 | 0.4 | √ | √ |
| | 90.6 | 4.0 | 500 | 10.6 | 35.1 | 32.7 | 0.9 | 0.3 | 30.3 | 2.3 | 51.1 | 0.0 | √ | √ |

| | | | | | | | | | | | | | | |
|---|---|---|---|---|---|---|---|---|---|---|---|---|---|---|
| | 90.6 | 4.0 | 2000 | 2.7 | 34.8 | 124.6 | 2.5 | 0.4 | 35.1 | 2.8 | 164.6 | 0.0 | √ | |
| E87-23 | 87.0 | 6.0 | 2 | 30.5 | 32.9 | 197.1 | 1.3 | 0.4 | 1.4 | 0.0 | 3.4 | 0.1 | √ | √ |
| | 87.0 | 6.0 | 50 (DCM) | 26.6 | 34.6 | 44.7 | 1.4 | 0.5 | 13.6 | 0.8 | 12.6 | 0.4 | √ | √ |
| | 87.0 | 6.0 | 500 | 10.1 | 35.0 | 21.2 | 1.4 | 0.4 | 37.0 | 2.7 | 68.6 | 0.0 | √ | √ |
| | 87.0 | 6.0 | 2000 | 2.9 | 34.8 | 116.7 | 1.3 | 0.4 | 38.0 | 2.9 | 171.4 | 0.0 | √ | |
| E87-30 | 87.0 | 13.0 | 2 | 30.4 | 32.1 | 202.3 | 2.7 | 0.1 | 1.4 | 0.2 | 4.6 | 0.1 | √ | √ |
| | 87.0 | 13.0 | 90 (DCM) | 24.8 | 34.7 | 45.7 | 0.0 | 0.6 | 0.4 | 0.6 | 5.9 | 0.3 | √ | √ |
| | 87.0 | 13.0 | 500 | 9.9 | 35.0 | 6.2 | 2.4 | 0.2 | 46.8 | 2.8 | 66.8 | 0.0 | √ | √ |
| | 87.0 | 13.0 | 2000 | 2.6 | 34.8 | 117.6 | 0.0 | 0.1 | 45.2 | 3.1 | 172.5 | 0.0 | √ | |
| E87-32 | 87.0 | 15.0 | 2 | 29.5 | 33.6 | 152.7 | 3.2 | 0.2 | 1.5 | 0.1 | 2.5 | 0.2 | √ | √ |

| | | | | | | | | | | | | | | |
|---|---|---|---|---|---|---|---|---|---|---|---|---|---|---|
| | 87.0 | 15.0 | 85 (DCM) | 28.0 | 34.1 | 187.9 | 1.2 | 0.2 | 1.9 | 0.3 | 2.6 | - | √ | √ |
| | 87.0 | 15.0 | 500 | 10.0 | 35.0 | 7.0 | 4.6 | 0.2 | 39.6 | 2.6 | 66.8 | 0.0 | √ | √ |
| | 87.0 | 15.0 | 2000 | 2.7 | 34.8 | 115.3 | 3.6 | 0.2 | 24.7 | 2.6 | 167.1 | 0.0 | √ | |
| Tara-068 | -31.0 | 4.6 | 722.7 | 7.0 | 34.5 | 195.2 | - | 0.0 | 22.1 | 1.5 | 12.1 | - | | √ |
| Tara-072 | -8.8 | -17.9 | 792.7 | 4.7 | 34.5 | 143.2 | - | 0.0 | 37.0 | 2.6 | 34.4 | - | | √ |
| Tara-078 | -30.2 | -43.3 | 792.7 | 5.9 | 34.4 | 214.8 | - | 0.0 | 28.2 | 2.0 | 13.9 | - | | √ |
| Tara-122 | -9.0 | -139.2 | 594.6 | 7.2 | 34.6 | 38.1 | - | 0.0 | 34.6 | 2.4 | 22.5 | - | | √ |
| Tara-138 | 6.4 | -103.1 | 444.9 | 8.2 | 34.6 | 0.8 | - | 0.0 | 36.2 | 3.2 | 52.9 | - | | √ |

**Table S2. Major properties of Molecular Ecological Network.**

| | nodes | links | $R^2$ of power-law | avgK | avgCC | avgGD | modularity | module |
|---|---|---|---|---|---|---|---|---|
| 2 m | 284 | 1357 | 0.786 | 9.556 | 0.236 | 3.592 | 0.445 | 9 |
| DCM | 317 | 1153 | 0.854 | 7.274 | 0.194 | 3.856 | 0.504 | 19 |
| 500 m | 219 | 156 | 0.947 | 1.425 | 0.049 | 2.450 | 0.958 | 72 |
| 2000 m | 336 | 575 | 0.824 | 3.423 | 0.153 | 7.098 | 0.702 | 59 |
| all | 116 | 469 | 0.764 | 8.086 | 0.414 | 2.334 | 0.297 | 8 |

avgK, average degree; avgCC, average clustering coefficient; avgGD, average path distance.

**Table S3. *p*-value of Analysis of Variance (ANOVA) showed the differences of some genes related to nitrogen metabolism between 2 m, DCM and 500 m in the BoB.** *p*-value are bold when the differences are significant ($p < 0.05$).

| Gene | 2 m vs. DCM | 2 m vs. 500 m | DCM vs. 500 m |
|---|---|---|---|
| *amoA* | **0.005** | **< 0.001** | **< 0.001** |
| *amoB* | **0.004** | **< 0.001** | **< 0.001** |
| *amoC* | **0.011** | **< 0.001** | **< 0.001** |
| *narGZ, nxrA1* | 0.05 | **< 0.001** | **< 0.001** |
| *nirD* | 0.098 | **0.046** | **0.002** |
| *nirB* | 0.104 | 0.692 | 0.171 |
| *narIV* | 0.441 | **< 0.001** | **< 0.001** |
| *narHY, nxrB* | 0.997 | **< 0.001** | **< 0.001** |
| *narGZ, nxrA2* | 0.815 | **0.005** | **0.003** |
| *nirK* | 0.067 | **< 0.001** | **< 0.001** |

**Table S4. *p*-value of Analysis of Variance (ANOVA) showed the differences of some genes related to sulfur metabolism between 2 m, DCM and 500 m in the BoB.** *p*-value are bold when the differences are significant ($p < 0.05$).

| Gene | 2 m vs. DCM | 2 m vs. 500 m | DCM vs. 500 m |
|---|---|---|---|
| *cysI* | 0.148 | **< 0.001** | **< 0.001** |
| *cysJ* | **0.003** | **0.019** | 0.247 |
| *sir* | 0.316 | **0.046** | **0.007** |
| *dsrA* | **0.017** | **< 0.001** | **< 0.001** |
| *dsrB* | **0.026** | **< 0.001** | **< 0.001** |

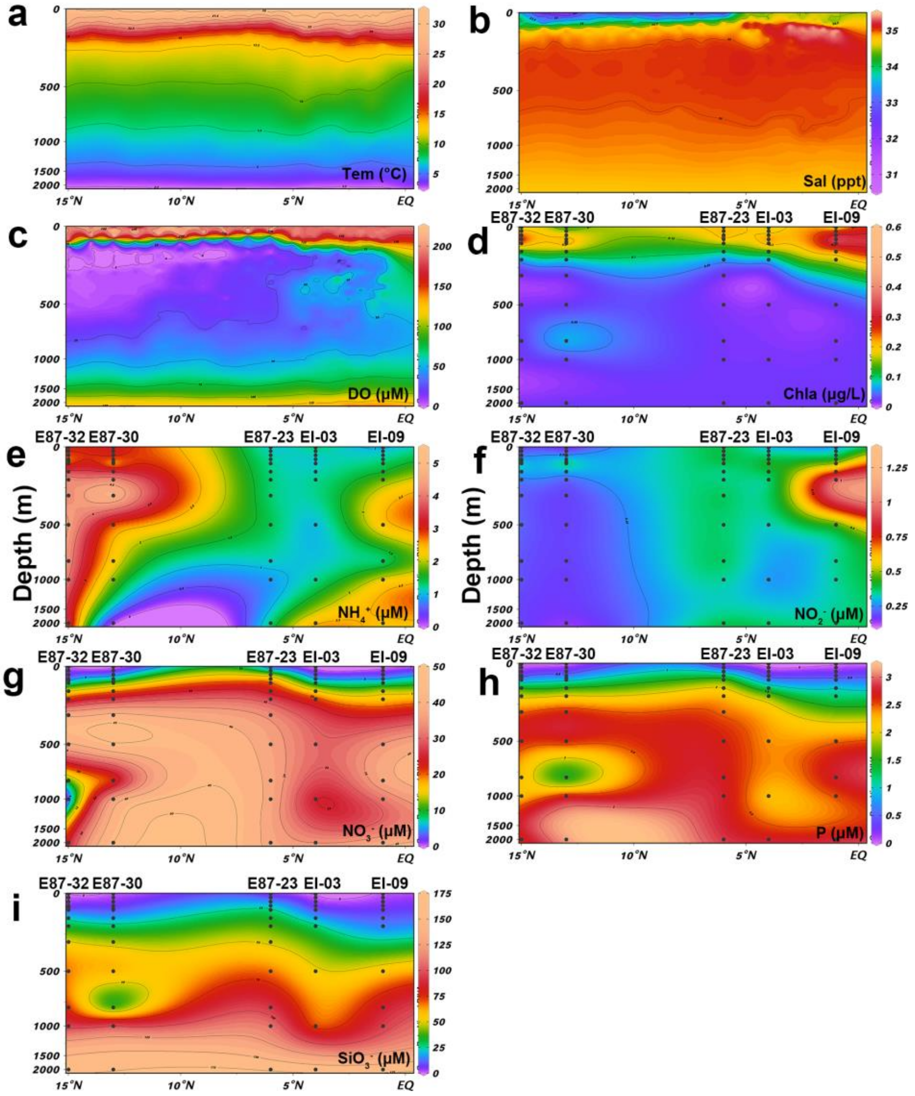


**Fig. S1. Vertical distributions along latitude of temperature (Tem, °C, a), salinity (Sal, b), dissolved oxygen (DO, μM, c), Chlorophyll *a* (Chl*a*, μg/L, d), $NH_4^+$ (μM, e), $NO_2^-$ (μM, f), $NO_3^-$ (μM, g), phosphate (P, μM, h), SiO3- (μM, i).** Temperature, salinity and DO were generated based on CTD sensor data, and the resolution of the data was per meter in vertical and per longitude in horizontal. Dots display the sampling stations and depths.

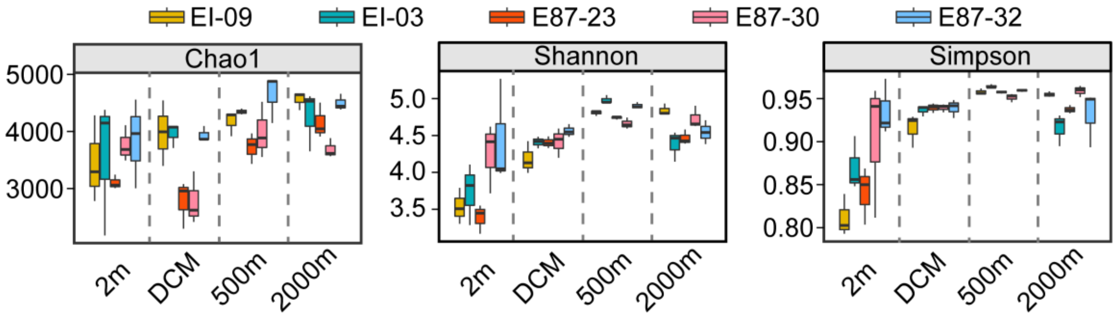


**Fig. S2. Alpha diversity, including Chao1, Shannon and Simpson index (based on 16S rRNA gene) of the prokaryotic community in the BoB.**

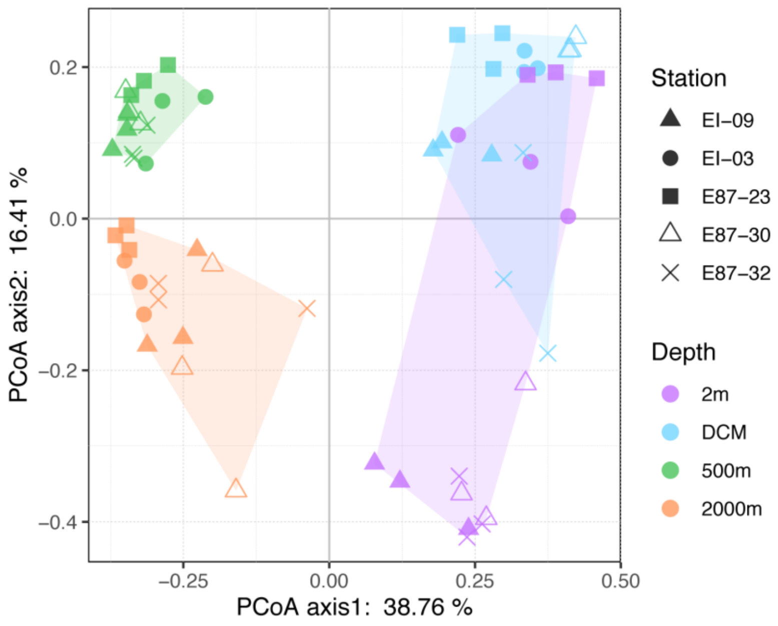


**Fig. S3. Principal coordinate analysis (PCoA) plot based on Bray Curtis dissimilarities of 16S rRNA gene for the prokaryotic community.**

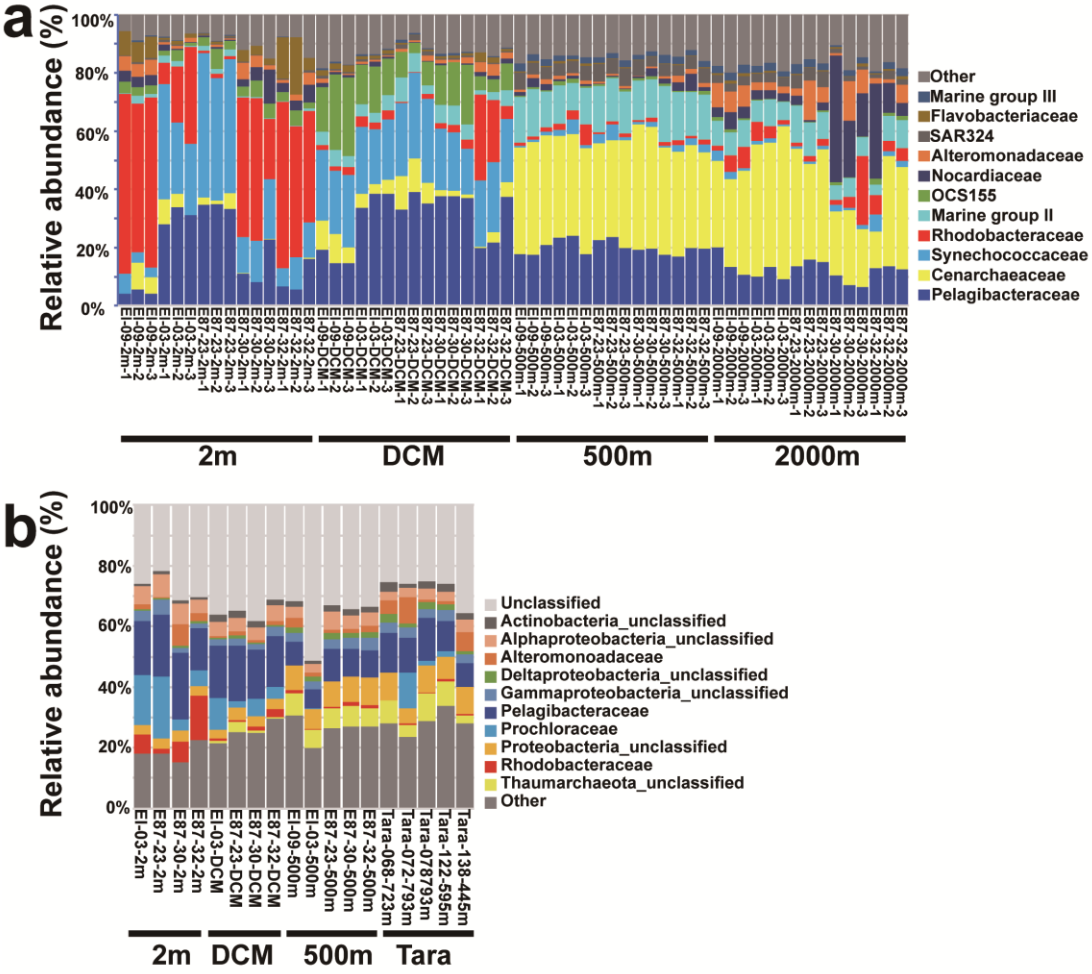


**Fig. S4. The prokaryotic community composition at the family level based on 16S rRNA dataset (a) and metagenome (b), respectively.**

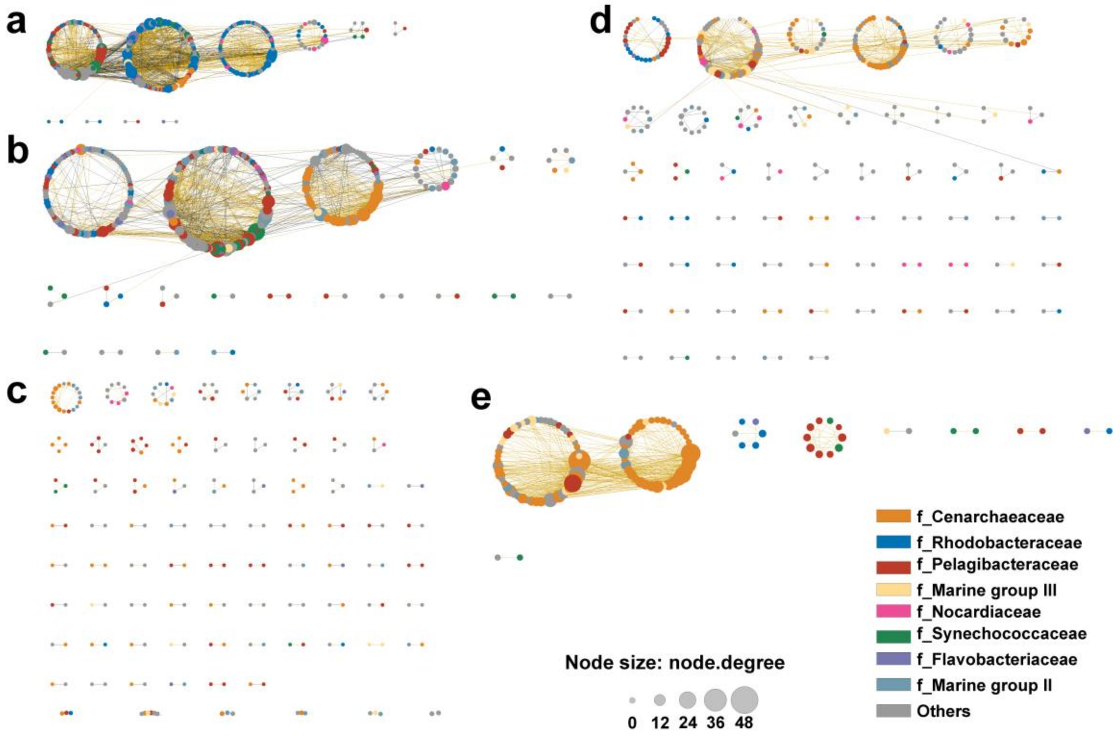


**Fig. S5. Co-occurrence network structures at 2 m (a), DCM (b), 500 m (c), 2000 m (d), all depths (e) of the BoB.** The networks are visualized with attribute circle layout based on the family level. The colors of the nodes indicate different family OTUs, as shown in the key at the right. A yellow line indicates a significant positive correlation between two OTUs (nodes), while a grey line indicates a significant negative correlation.

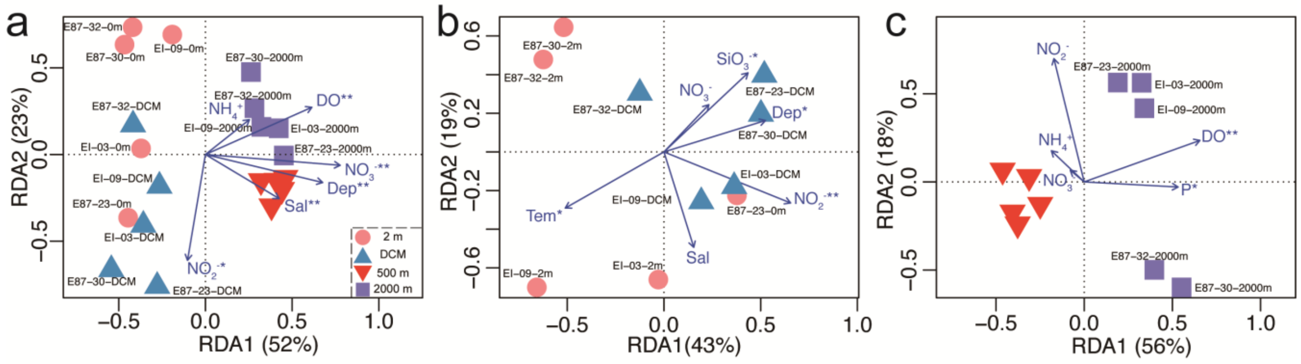


**Fig. S6. RDA ordinations showing community composition (based on 16S rRNA gene) in relation to environmental variables in the all depth (a), euphotic zones (5 m and DCM, b) and deep ocean (500 m and 2000 m, c) of the BoB.**

Multicollinearity was tested by the variance inflation factor (VIF) and the variables with a VIF > 10 were removed. "*" indicates the significant correlation (**, $p < 0.01$; *, $p < 0.05$) between environmental parameters and prokaryotic community composition.

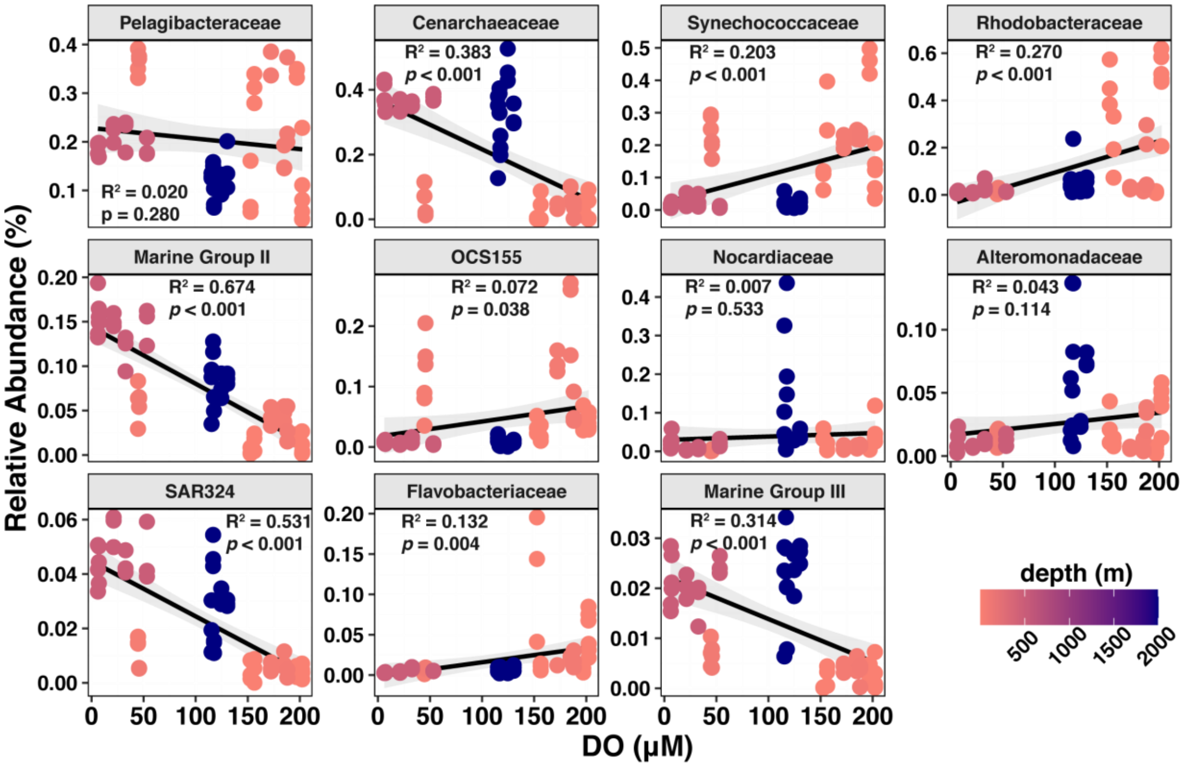


**Fig. S7. Linear correlations between DO concentration and the relative abundance of the top 11 prokaryotic families.** The grey shadow indicates the confidence interval.
